# Effectiveness of clarithromycin in patients with yellow nail syndrome

**DOI:** 10.1186/s12890-018-0707-4

**Published:** 2018-08-15

**Authors:** Sachi Matsubayashi, Manabu Suzuki, Tomoyuki Suzuki, Ayako Shiozawa, Konomi Kobayashi, Satoru Ishii, Motoyasu Iikura, Shinyu Izumi, Koichiro Kudo, Haruhito Sugiyama

**Affiliations:** 1 Department of Respiratory Medicine, National Center for Global Health and Medicine, 1-21-1 Toyama Shinjuku-ku, Tokyo, 162-8655 Japan; 2 Waseda University Organization for Regional and Inter-regional Studies, 2-1-1 Nishi-Shinjuku, Shinjuku-ku, Tokyo, 169-0051 Japan

**Keywords:** Bronchiectasis, Clarithromycin, Signs and symptoms, respiratory, Yellow nail syndrome

## Abstract

**Background:**

Yellow nail syndrome (YNS) is a rare disease characterized by the triad of thickened, slow-growing yellow nails, lymphedema, and chronic respiratory manifestations. The cause of YNS is not known; however, it is suggested to be due to a congenital lymph abnormality. Since YNS is accompanied by chronic bronchial infection in more than half of patients, we hypothesized that treatment with clarithromycin (CAM) could be effective. We therefore evaluated the effectiveness of CAM against nail discoloration and respiratory manifestation in patients with YNS.

**Methods:**

We conducted an observational study involving 5 patients with YNS who were treated at our institution between January 2005 and January 2016. CAM was prescribed for every patient. Patient demographic information, comorbidities, medications, chest radiographs, and clinical data such as nail color were extracted to evaluate clinical outcome.

**Results:**

Mean patient age was 71.6 years, and 2 patients (40%) were male. Four patients had sinusitis, and 2 had rheumatoid arthritis. Regarding respiratory manifestations, 4 patients had sinobronchial syndrome and 2 had pleural effusion. Nail discoloration improved in every patient after CAM treatment. Four patients also experienced improvement in their respiratory manifestations.

**Conclusions:**

In patients with YNS, the anti-inflammatory activity of macrolides might improve their systemic inflammation. This improvement could help to reduce lymphedema and promote nail growth.

**Trial registration:**

Ethical approval was provided by the institutional review board of the National Center of Global Health and Medicine (NCGM-G-002143-00), in January 2017. This study is retrospectively registered for UMIN Clinical Trial Registry (UMIN000028514) in August 4th, 2017.

## Background

Yellow nail syndrome (YNS), a rare disease first described in 1964 by Samman and White [1], is characterized by yellow nails due to nail growth delay and lymphedema. Since Emerson’s 1966 report describing respiratory complications of the disease, YNS has been defined by the classical triad of yellow nails, lymphedema, and chronic respiratory manifestations [2]. A diagnosis of YNS can be based on the presence of 2 of the above 3 symptoms [3]. According to previous studies, only half of patients with YNS have all 3 conditions [4]. The etiology of YNS remains undefined, and there is no consensus about treatment strategy for this syndrome [5].

Yellow nails are the main clinical manifestation leading to a diagnosis of YNS. Nail discoloration (varying from pale yellow to dark green), hyperkeratosis, and onycholysis may occur in many patients. The nail grows more slowly than 0.2 mm/week compared with the minimum of 0.5–1.2 mm/week in healthy subjects [2]. These nail changes are thought to be an abnormality of growth. Defective lymphatic drainage around nails, usually congenital [1], microvasculopathy with protein leakage [4], accumulation of lipofuscin pigment [6], and titanium ion involvement [7] might be considered causes of nail abnormality.

Oral or topical vitamin E [8], antifungals [9], zinc [10], and topical corticosteroid plus active vitamin D3 [11] have been reported to be effective for treating yellow nails.

In more than half of patients, YNS is accompanied by chronic bronchial infection [5]. Clarithromycin (CAM) is generally effective for chronic lower respiratory tract infections. We hypothesized that CAM could also be an effective treatment for YNS, improving respiratory and nail manifestations through lymphatic drainage and anti-inflammatory effects. Some reports have shown that improvement of nail abnormalities corresponds to better control of respiratory manifestations [12]. We therefore analyzed the effectiveness of CAM against nail discoloration in patients with YNS.

## Methods

We conducted an observational study at the National Center of Global Health and Medicine (NCGM), a general hospital of the National Research and Development Agency in Tokyo, Japan, which has more than 700 inpatient beds. Eligible patients were aged 18 years or older, had been diagnosed with YNS, and were treated at NCGM between January 2005 and January 2016. All patients had been hospitalized because of exacerbation of lower respiratory infection, and were then diagnosed as having YNS with respiratory manifestations and nail discoloration. All patients received oral CAM. Patient demographic information, comorbidities, medications, chest radiographs, and clinical data such as nail color were collected to evaluate clinical outcomes. The primary outcome was improvement of nail color, thickness and onycholysis and the secondary outcome was control of respiratory manifestations. We assessed respiratory manifestations by comparing chest X-ray findings, such as consolidation and pleural effusion), and oxygen demand. All X-rays were independently reviewed by the same two experienced observers. Responses to CAM treatment were categorized as complete response (CR), partial response (PR), minor response (MR), and no response (NR). CR was defined as complete improvement of all fingernails. MR was defined as slightly improvement in less than half of the fingernails. PR was defined as intermediate improvement between CR and MR. Last, NR was defined as no change in the nails.

## Results

Five patients with YNS were included in the study. The characteristics and clinical presentation of each patient at treatment initiation are shown in Table 1. Mean patient age was 71.6 years (range 58–80 years), and 2 patients (40%) were male. In terms of comorbidities, 4 patients had sinobronchial syndrome and 2 had rheumatoid arthritis (RA).

**Table 1:** Characteristics of patients at treatment initiation

	Age (years)	Sex	Comorbidities	Primary signs	Triad	Nail Manifestation	Medication
Patient 1	80	Female	SBS	Fever, cough	Bronchiectasis, yellow nails, leg edema	Yellow-green Thickening Onycholysis	Antifungal drug
Patient 2	67	Male	SBS, RA	Dyspnea, cough	Pleural effusion, yellow nails	Yellow Thickening	Bucillamine, predonisolone, SASP, FK-506
Patient 3	58	Female	Duodenal ulcer	Dyspnea, cough	Bronchiectasis, yellow nails, periorbital edema	Whitish-yellow Thickening	Antifungal drug
Patient 4	70	Male	SBS	Fever, cough	Bronchiectasis, yellow nails	Yellow Thickening	None
Patient 5	80	Female	SBS, RA	Dyspnea, leg swelling	Pleural effusion, yellow nails, leg edema	Yellow Thickening	Bucillamine

*RA* rheumatoid arthritis, *SASP* salazosulfapyridine, *SBS* sinobronchial syndrome

**Fig. 1:**
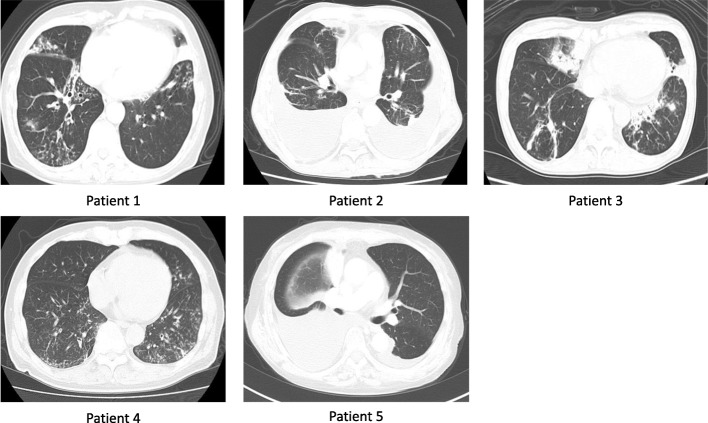
Initial computed tomography chest images of each patient

Based on examination by dermatologists, two patients (Patients 1 and 3) had taken antifungal drugs on suspicion of candida paronychia infection, but their nails had not responded to the treatment. Both patients with RA (Patients 2 and 5) had pleural effusion; the others had bronchiectasis as a respiratory manifestation. The patients with RA had taken bucillamine for RA treatment before being diagnosed with YNS. In terms of the nails, all five patients had nail discoloration (varying from whitish-yellow to yellow-green) and thickening. And patient 1 had onycholysis. Initial chest computed tomography scans of each patient are shown in Fig. 1.

**Fig. 2:**
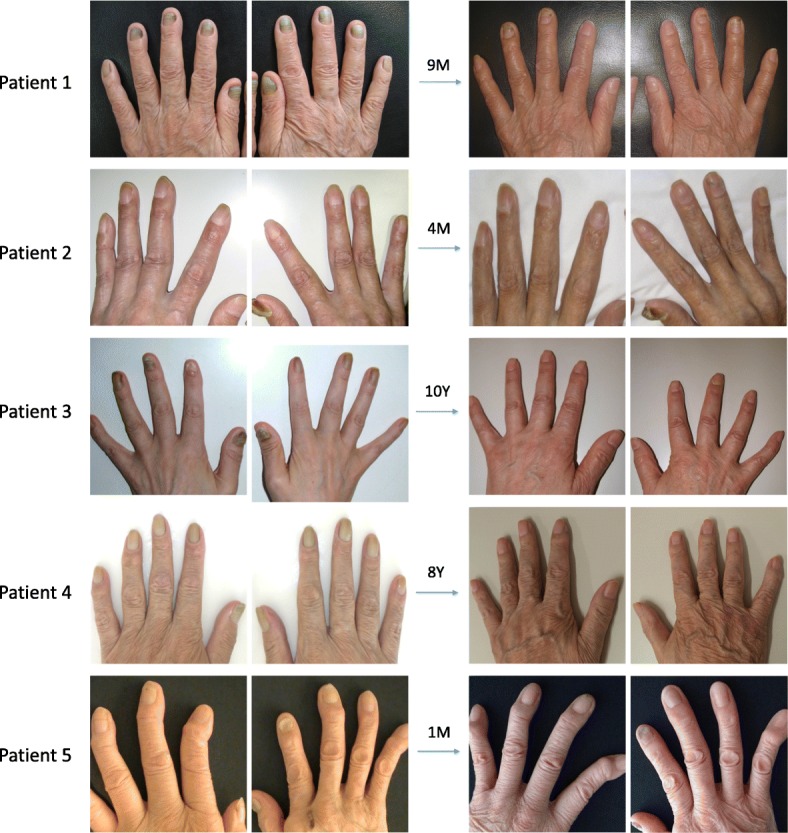
Nail discoloration of each patient before and after clarithromycin treatment. *Y: years; M: months

The clinical course and treatment for each patient are shown in Table 2. CAM was prescribed for every patient. Two patients began with 400 mg/day of CAM; the others began with 200 mg/day, but nail discoloration did not improve until the dosage was increased to 400–600 mg/day. The final CAM dosage for every patient was 400 mg/day. Improvement of nail discoloration was seen in every patient after CAM treatment (Fig. 2), with the time from treatment initiation to nail color improvement ranging widely (1 month to 2.5 years).

**Table 2:** Clinical course and treatment

	CAM dosage (mg/day)	Nail discoloration	Time to nail improvement	Time to best nail improvement	Respiratory manifestations
Patient 1	400	Improved, all fingers	1 month	9 months (CR)	Improved
Patient 2	200 ➔400 (4 months)	Slightly improved (index and middle fingers)	4 months on 400 mg/day	4 months (MR)	Uncontrolled
Patient 3	200 ➔600 (2 years), then 400 (3 years)	Improved, all fingers	2.5 years on 600 mg/day	10 years (CR)	Improved
Patient 4	200 ➔600 (2 years), then 400 (4 years)	Improved, all fingers	3 years on 600 mg/day	4 years (CR)	Improved
Patient 5	400	Improved, all fingers	1 month	5 months (PR)	Improved

*CAM* clarithromycin

**Fig. 3:**
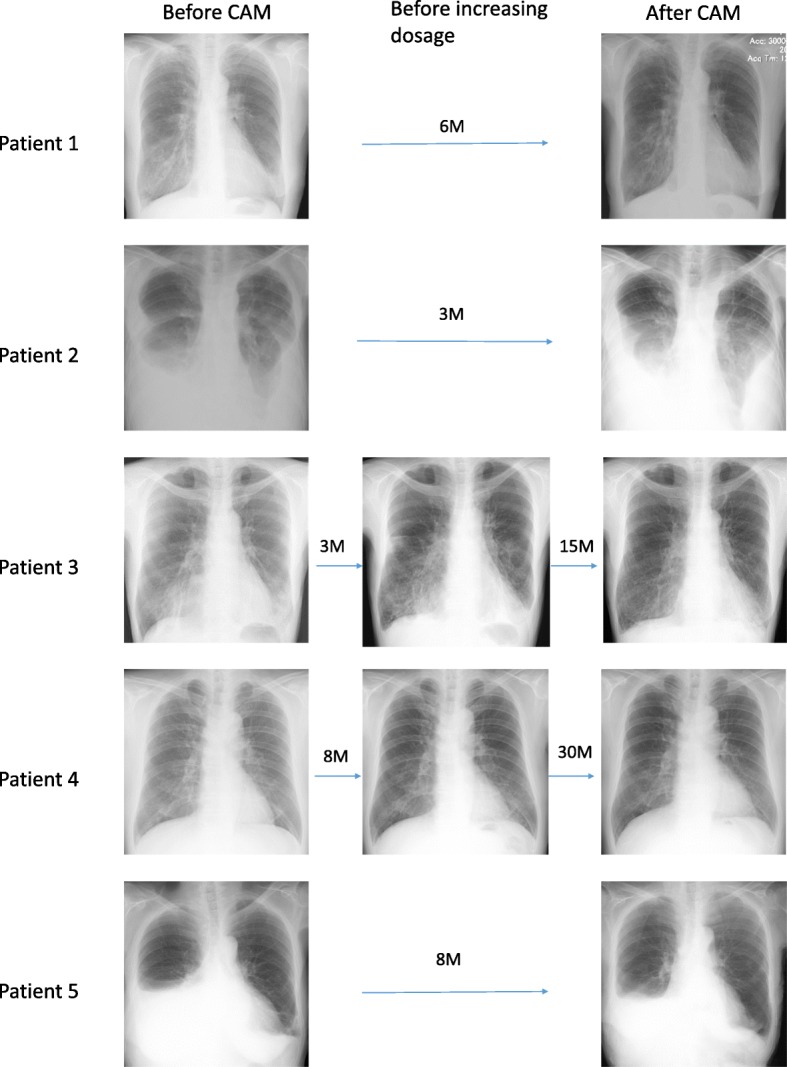
Chest radiographs of each patient before and after clarithromycin treatment. *Y: years; M: months

Regarding the secondary outcome, respiratory manifestations of YNS improved in parallel with nail improvement in 4 patients (Fig. 3). Three patients (Patient 1, 3, and 4) showed CR to CAM treatment. In patient 2, nails improved just slightly; categorized as MR. Only about half nails were improved in Patient 5.

In Patient 2, pleural effusion was uncontrollable. In contrast to bronchial manifestations such as cough and phlegm, pleural effusion management was quite difficult. We inserted a drainage tube into the chest space, but the lung was not fully expansible.

Two patients (Patient 2 and 5) died during CAM treatment; Patient 2 died of a respiratory disorder 1 year after CAM treatment initiation, and Patient 5 died of a urinary tract infection 6 months after CAM treatment initiation.

## Discussion

YNS is a rare disease characterized by the triad of thickened, slow-growing yellow nails, lymphedema, and chronic respiratory manifestations, including pleural effusion, bronchiectasis, rhinosinusitis, and recurrent lung infections [2]. According to Hiller’s definition, the presence of 2 of these 3 symptoms is sufficient for a diagnosis of YNS [3]. Individual manifestations of the syndrome can appear at different times, even with an interval of several years. All 3 conditions coexist in only 27–60% of patients with YNS [4, 13]. Eighty-nine percent of patients have nail changes, 80% have lymphedema, and 40–68% have exudative pleural effusion [11]. From 25 to 75% of patients have chronic sinusitis or bronchiectasis [5].

The etiology of YNS remains undefined, but may be associated with congenital lymph abnormality [1], microvasculopathy, and protein leakage [4]. Stresses such as infection worsen lymph drainage impairment, which can lead to lymphedema. Bronchial lymphedema can induce bronchiectasis, and lymphedema around the nails can reduce the rate of nail growth by more than 90% [1], inducing thickening and yellowing. Some studies have also suggested that YNS may occur secondary to environmental or iatrogenic exposures [7, 12, 14].

There is no consensus about treatment strategy for YNS. We previously reported a case of yellow nail improvement using only CAM [15]. Other treatment options include topical vitamin E, which may prevent nail oxidation [8], zinc [10] and topical corticosteroid plus active vitamin D3 [11], which has been reported to be effective for treating yellow nails. The respiratory symptoms of YNS are the most fatal [10, 11]. Treatment with octreotide [16–18], pleurodesis [19, 20], and shunt replacement are performed for management of pleural effusion [21–23].

CAM is a 14-membered macrolide. These drugs have both anti-inflammatory and antibacterial effects due to their inhibition of epithelial secretion of water and mucus [24] and production of inflammatory cytokines. For example, this drug class has been found to decrease secretion of interleukin (IL)-6 and granulocyte macrophage colony-stimulating factor from epithelial cells; inhibit production of IL-2, IL-3, and IL-4 by lymphocytes; and decrease production of IL-1, tumor necrosis factor alpha, and IL-8 by monocytes and macrophages [25]. A previous study showed the importance of CD4+ T cells and their cytokines in the pathology of lymphedema. And tacrolimus, which has macrolide structure, significantly increased lymphangiogenesis by regulating T-cell inflammatory response and expression of anti-lymphangiogenic growth factors [26].

We used CAM for the following reasons. First, erythromycin has more side effects than CAM. Second, we could only prescribe azithromycin for 3 days according to the health regulations in our country.

We suggest that the anti-inflammatory activity of CAM contributes to decreased lymphedema around the nails, thereby improving their color, while decreased mucus secretion improves respiratory symptoms. In our patients, improvement of nail discoloration generally corresponded to better control of respiratory manifestations. Bronchial symptoms such as cough and phlegm could be managed due to use of CAM, but pleural effusion was difficult to manage.

Some previous studies have showed that the anti-inflammatory properties of these drugs may be dose dependent [27, 28]. The results of the present study also suggest dose dependence. Because some patients showed improvement only after increasing CAM from 200 mg to 400 mg daily, we recommend a dosage of more than 400 mg/day.

This was an observational, single-arm study. Because of the rarity of YNS, it would be difficult to perform a randomized controlled trial. It is also difficult to compare differences in nail manifestations objectively, so we categorized treatment responses as used in anti-cancer therapy, such as CR and PR. Furthermore, we cannot exclude the possibility that some of the observed lymphedema and respiratory manifestations were related either to other medications or to RA. Patient 2 had stopped taking bucillamine 6 months prior to CAM start, and confirmed that the nail and respiratory manifestations had not improved. But patient 5 started taking CAM only after bucillamine was stopped. In fact, improvement of nail and respiratory symptoms was relatively weaker in patients with RA. This study has revealed a large variation in the time taken to achieve the primary outcome. We could not deny spontaneous improvement. But few cases of spontaneous recovery have been reported previously. We cannot conclusively verify that CAM is useful for YNS as a treatment through an observational study, but we could clinically suggest an association between CAM and improvement of clinical manifestations (at both nail and respiratory level). Further investigations are needed to predict factors affecting treatment response.

## Conclusion

To our knowledge, this is the first study showing improvement of nail discoloration and respiratory manifestations in patients with YNS treated with only CAM.

In patients with YNS, the anti-inflammatory activity of macrolides is likely associated with improvement of systemic inflammation. This improvement could help reduce lymphedema and promote nail growth. Further studies are needed to confirm our result.

## Abbreviations

CAM: Clarithromycin
IL: Interleukin
NCGM: National Center of Global Health and Medicine
RA: Rheumatoid arthritis
YNS: Yellow nail syndrome

## References

[1] Samman PD, White WF (1964). The yellow nail syndrome. Br J Dermatol.

[2] Emerson PA (1966). Yellow nails. Lymphedema, and pleural effusions. Thorax.

[3] Hiller E, Rosenow EC, Olsen AM (1972). Pulmonary manifestation of the yellow nail syndrome. Chest.

[4] Maldonado F, Tazelaar HD, Wang C, Ryu JH (2008). Yellow nail syndrome: analysis of 41 consecutive patients. Chest.

[5] Valdés L, Huggins JT, Gude F, Ferreiro L, Alvarez-Dobaño JM, Golpe A, Toubes ME, González-Barcala FJ, José ES, Sahn SA (2014). Characteristics of patients with yellow nail syndrome and pleural effusion. Respirology.

[6] Norton L (1985). Further observations on the yellow nail syndrome with therapeutic effects of oral alpha-tocopherol. Cutis.

[7] Berglund F, Carlmark B (2011). Titanium, sinusitis, and the yellow nail syndrome. Biol Trace Elem Res.

[8] Ayres S, Mihan R (1973). Yellow nail syndrome: response to vitamin E. Arch Dermatol.

[9] Tosti A, Piraccini BM, Iorizzo M (2002). Systemic itraconazole in the yellow nail syndrome. Br J Dermatol.

[10] Arroyo JF, Cohen ML (1993). Improvement of yellow nail syndrome with oral zinc supplementation. Clin Exp Dermatol.

[11] Nordkild P, Kromann-Andersen H, Struve-Christensen E (1986). Yellow nail syndrome-the triad of yellow nails, lymphedema and pleural effusions. A review of the literature and a case report. Acta Med Scand.

[12] Nakagomi D, Ikeda K, Hirotoshi K, Kobayashi Y, Suto A, Nakajima H (2013). Bucillamine-induced yellow nail in Japanese patients with rheumatoid arthritis: two case reports and a review of 36 reported cases. Rheumatol Int.

[13] Mambretti-Zumwalt J, Seidman JM, Higano N (1980). Yellow nail syndrome: complete triad with pleural protein turnover studies. South Med J.

[14] Banta DP, Dandamudi N, Parekh HJ, Anholm JD (2009). Yellow nail syndrome following thoracic surgery: a new association?. J Postgrad Med.

[15] Suzuki M, Yoshizawa A, Sugiyama H, Ichimura Y, Morita A, Takasaki J, Naka G, Hirano S, Izumi S, Takeda Y, Hojo M, Kobayashi N, Kudo K (2011). A case of yellow nail syndrome with dramatically improved nail discoloration by oral clarithromycin. Case Rep Dermatol.

[16] Iqbal M, Rossoff LJ, Marzouk KA, Steinberg HN (2000). Yellow nail syndrome: resolution of yellow nails after successful treatment of breast cancer. Chest.

[17] Hillerdal G (2007). Yellow nail syndrome: treatment with octreotide. Clin Respire J.

[18] Widjaja A, Gratz KF, Ockenga J, Wagner S, Manns MP (1999). Octreotide for therapy of chylous ascites in yellow nail syndrome. Gastroenterology.

[19] Jiva TM, Poe RH, Kallay MC (1994). Pleural effusion in yellow nail syndrome: chemical pleurodesis and its outcome. Respiration.

[20] Yamagishi T, Hatanaka N, Kamemura H, Nakazawa I, Hirano Y, Kodaka N, Miura A, Kitahara A, Sawata T, Hosaka K, Sanno K (2007). Idiopathic yellow nail syndrome successfully treated with OK-432. Intern Med.

[21] Tidder J, Pand CL. A staged management of prolonged chylothorax in a patient with yellow nail syndrome. BMJ Case Rep. 2012; 10.1136/bcr-2012-006469. 10.1136/bcr-2012-006469 PMC4544095 23125296

[22] Moorjani N, Winter RJ, Yigsaw YA, Maiwand MO (2004). Pleural effusion in yellow nail syndrome: treatment with bilateral pleuro-peritoneal shunts. Respiration.

[23] Tanaka E, Matsumoto K, Shindo T, Taguchi Y (2005). Implantation of pleurovenous shunt for massive chylothorax in a patient with yellow nail syndrome. Thorax.

[24] Ikeda K, Wu D, Takasaka T (1995). Inhibition of acetylcholine-evoked cl-currents by 14-membered macrolide antibiotics in isolated acinar cells of the Guinea pig nasal gland. Am J Respir Cell Mol Biol.

[25] Morikawa K, Oseko F, Morikawa S, Iwamoto K (1994). Immunomodulatory effects of three macrolides, midecamycin acetate, josamycin, and clarithmycin, in human T-lymphocyte function in vitro. Antimicrob Agents Chemother.

[26] Gardenier JC, Kataru RP, Hespe GE, Savetsky IL, Torrisi JS, Nores GD, Jowhar DK, Nitti MD, Schofield RC, Carlow DC, Mehrara BJ. Topical tacrolimus for the treatment of secondary lymphedema. Nat Commun. 2017; 10.1038/ncomms14345. 10.1038/ncomms14345 PMC5309859 28186091

[27] Zarogoulidis P, Pananas N, Kioumis I, Chatzaki E, Maltezos E, Zaroguldis K (2012). Macrolides; from in vitro anti-inflammatory and immunomodulatory properties to clinical practice in respiratory disease. Eur J Clin Pharmacol.

[28] Ianaro A, Ialenti A, Maffia P, Sautebin L, Rombola L, Carnuccio R, Iuvone T, D’Acquisto F, Di Rosa M (2000). Antiinflammatory activity of macrolide antibiotics. J Pharmacol Exp Ther.

